# Medicines in pregnancy

**DOI:** 10.12688/f1000research.17535.1

**Published:** 2019-06-20

**Authors:** Sarah JE Stock, Jane E Norman

**Affiliations:** 1 Usher Institute of Population Health Sciences and Informatics, University of Edinburgh, Nine Edinburgh BioQuarter, 9 Little France Road, Edinburgh, EH16 4UX, UK; 2 MRC Centre for Reproductive Health, University of Edinburgh, Edinburgh BioQuarter, Edinburgh, EH16 4SA, UK; 3 Faculty of Health Sciences, University of Bristol, 5 Tyndall Avenue, Bristol, UK

**Keywords:** Pregnancy, Pharmacokinetics, Pharmacovigilance, Medicines, Public Health, Maternal Health, Newborn Health, Child Health

## Abstract

Medicine use in pregnancy is extremely common, but there are significant knowledge gaps surrounding the safety, dosage and long-term effects of drugs used. Pregnant women have been purposively excluded from clinical trials of the majority of treatments for conditions that may occur concurrently with pregnancy. There is minimal information on the pharmacokinetics of many existing treatments and no systematic capture of long-term outcome data to help inform choices. Treatments commonly used in pregnancy are thus often old and untested, not optimised in dose, and prescribed off-label without adequate safety information. In addition, there has been a staggering lack of investment in drug development for obstetric conditions for decades. This is a major public health concern, and pregnancy complications are the leading cause of mortality in children under five years old globally, and health in pregnancy is a major determinant of women’s long-term health and wellbeing. There is an acute need for adequate investment and legislation to boost inclusion of pregnant women in clinical studies, capture high-quality information on medication use in pregnancy in general, and encourage new medicinal product development for obstetric conditions.

## Introduction

In high-income countries, four out of five pregnant women are prescribed one or more medications in pregnancy ^1^, and even higher levels of pregnant women self-medicate with over-the-counter preparations ^2^. Medicines may be taken for a wide range of acute or non-acute indications, as outlined in Box 1. However, more than 98% of drugs have insufficient pharmacokinetic or safety data to guide dosing in women who are pregnant or breastfeeding ^3, 4^.

**Box 1.** Potential indications for medicine use in pregnancy and examples of types of treatments

Indication	Examples of treatments
Medical conditions that predate, develop during, or are recognised for the first time in pregnancy	Asthma treatments, treatments for thyroid disorders, anti- hypertensives, insulin, anti- epileptics, anti-retrovirals
Psychiatric conditions that predate, develop during, or are recognised for the first time in pregnancy	Anti-depressants and anxiolytics, mood stabilisers
Replacement therapies	Methadone, nicotine replacement
Symptoms caused by or exacerbated by pregnancy	Anti-emetics, laxatives, antacids
Intercurrent illnesses	Anti-infectives, analgesics
Vaccinations	Pertussis, influenza
Fertility and miscarriage treatments	Clomiphene, progesterone, low-molecular-weight heparin
Nutritional supplementation and vitamins	Iron supplements, folic acid
Prevention and treatment of obstetric conditions (pre- term labour, pre-eclampsia, fetal growth restriction, gestational diabetes)	Aspirin, progestagens, anti- hypertensives, tocolytics, hypoglycaemic agents
Treatments to optimise the health of the newborn	Antenatal corticosteroids, magnesium sulphate

Very few drugs have been optimised for use by pregnant mothers, and there has been chronic under-investment both in new drug development and in clinical trials of established therapies in pregnancy ^5, 6^. Even when teratogenic effects of medications are recognised (as in the case for sodium valproate), messages about risk may not be delivered effectively to women of reproductive age ^7^ and it can take decades for legislation to be enacted to support safe prescribing ^8^. Research into medication in pregnancy is complicated by a number of factors that we outline below. Nevertheless, technologies are available that, if appropriately applied, have the potential to greatly improve the delivery of safe and efficacious medications in pregnancy.

## Gaps in knowledge on medications in pregnancy

It is generally expected that the efficacy, optimal dose and any short-term harmful effects of medications will be determined in the pre-clinical and clinical trial phases of drug development. However, involvement of pregnant participants in drug studies has been negligible over the past decades and this is due to purposive exclusion because of teratogencity risks or other harmful effects on the fetus. Thus, safety information is rarely available for medications that are used in pregnancy ^5^. Physiological changes in pregnancy significantly affect the pharmacokinetics of prescribed drugs ( Box 2) and pregnancy can alter the course and symptomology of illnesses. The effectiveness of drugs may therefore be very different in pregnancy than without pregnancy ^5^. The scale of the evidence gap for appropriate dosing of medications in pregnant women was shown in a 2014 report that found that only 1.3% of pharmacokinetic studies were performed in pregnancy ^3^. The fact that less than 10% of these studies were industry-funded is evidence of the lack of engagement from pharmaceutical companies to provide these data and of the lack of incentives for them to do so. Even when changes in pharmacokinetics are recognised, there is a lack of studies describing how these impact outcomes of the mother and baby ^9^ and so the clinical implications of pharmacokinetic changes are unknown. Inadvertent under-treatment may contribute to maternal and perinatal morbidity associated with medical conditions in pregnancy.

**Box 2.** Physiological changes in pregnancy and potential effects on pharmacokinetics (as reviewed in 10)

Physiological change in pregnancy	Potential effect on pharmacokinetics of drugs
↓ gastric emptying/small bowel motility	↑ time to reach peak levels
↑ gastric pH	↓ absorption
↑ vascularity and oedema respiratory mucosa	↑ absorption of inhaled drugs
↑ minute ventilation	↓ protein binding due to respiratory alkalosis
↑ total body water, blood volume and capillary hydrostatic pressure	↑ volume of distribution of hydrophilic drugs
↑ glomerular filtration rate	↑ renal clearance
↓ serum albumin	↑ active fraction of drug
↑ CYP450 and ↑ UGT activity	↑ metabolism

In addition to differing pharmacokinetics, a key consideration is the potential effect of treatments on the baby. Although genotoxicity, non-clinical reproduction and embryo-fetal developmental toxicity studies are standard during the development of most drugs, variations in species-specific effects can limit the ability of pre-clinical studies to predict human teratogenesis ^5^. Deliberate exclusion of pregnant women from clinical trials of medicinal products means that phase 2 or 3 trial data in pregnancy are virtually non-existent for drugs that are not one of the handful of agents designed for a specific obstetric indication. This has two important implications for the care of pregnant women. First, a large number of medications have contraindications or special warnings because they have not been sufficiently studied in pregnancy, limiting the availability of potentially effective treatments to pregnant women. The teratogenic risk of 168 of 172 medications approved by the US Food and Drug Administration (FDA) between 2000 and 2010 was found to be undetermined, and no data about the risk in pregnancy were available for 126 of them (73%) ^4^. This means that pregnant women may be unnecessarily denied the opportunity to receive medicines that could improve their health or the health of their baby. Second, establishing the safety of medications is nearly exclusively dependent on data collected post-authorisation from pregnant women who are inadvertently exposed or in whom the risks of stopping treatment are perceived to outweigh any potential risks to the baby ^11^.

## Challenges in pharmacovigilance

A common source of post-authorisation safety data on medicines in pregnancy consists of reports from pregnancy exposure registries. These have variously been established by pharmaceutical companies, academic groups and regulatory authorities. They have been used to provide reassurance that certain medications are not major teratogens and to identify signals of teratogenicity that require further investigation ^12^. However, although they may detect signals of high-risk teratogenesis (where around 25% of exposed babies are affected), they are generally under-powered to investigate moderate teratogenicity (for example, a 2- to 10-fold increase in risk) ^11^. Numbers of participants are limited by low levels of enrolment, which is voluntary, and loss to follow-up of registered participants. For example, none of the five prospective pregnancy registries sponsored by the pharmaceutical company GlaxoSmithKline (Brentford, UK) achieved enrolment of 1000 pregnancies in the first 10 years of medication marketing, the number considered by the European Committee for Medicinal Products for Human Use to be representative of widespread exposure ^13^. Lack of an untreated comparator group can also be problematic, making it difficult to distinguish the effects of a treatment for a disease from the effect of the disease process itself ^12^.

In order to more fully determine risks and benefits of medications in pregnancy, large and complex datasets are required. Pregnant women take medications for a variety of reasons. A proportion of women who become pregnant are already on treatment for pre-existing medical conditions, and pregnancy symptoms (such as indigestion or nausea and vomiting), concurrent illnesses or infections, and obstetric complications (such as pre-eclampsia, intrahepatic cholestasis of pregnancy or pre-term labour) can precipitate new medicine use. The range of indications for treatment means that, although prescriptions are common, use of individual drugs can be comparatively rare ^14^, necessitating large and diverse data sources to study treatment effects ^5, 15^. Furthermore, many over-the-counter remedies used by pregnant women are not routinely recorded. Accurate information about pregnancy exposures and potential confounding factors (including other medicine use) is required to allow appropriate risk assessment.

A further complexity is the large number of effects that medications can have. Teratogenesis is often perceived as being synonymous with structural malformations, but pregnancy medications have a broad range of potential effects in addition to birth defects, and these may vary depending on the drug, the dose and the period of exposure ^16^. Potential impacts include increasing rates of miscarriage, stillbirth, fetal growth perturbations, and pre-term birth. Evaluation of pregnancy treatments thus needs to be broad, include women whose pregnancies have not continued past the first trimester (to capture effects on early pregnancy complications and miscarriage), and be of sufficient scale to recognise rare but serious events such as stillbirth. Methods should be sufficiently sensitive to detect severe disabilities but also more subtle effects. For example, drugs may cause neurodevelopmental disability but also have effects on behaviour and educational attainment which may not be captured in standard medical records. Long-term follow-up is essential as adverse effects may not be recognised until adulthood or beyond or indeed until the second generation. For example, diethylstilboestrol increased the risk of vaginal adnenocarcinoma in *in utero*–exposed offspring and has been associated with health effects in subsequent generations ^17^.

## Opportunities for advancing knowledge

Although study of medicines in pregnancy is challenging, barriers should not be seen as insurmountable, and advances in methodology provide opportunities for new knowledge. Pre-clinical studies of the safety profile of medications should form the basis of any initial studies of any new agent that has the potential to be used in pregnancy ^5^. Recommendations regarding the species used in drug safety testing are currently lacking. However, as there are huge interspecies differences in both placentation and duration of gestation, guidelines are needed to ensure that safety testing regarding relevance to human pregnancy is optimised. Pre-clinical testing should also ideally include follow-up of an entire generation through a complete reproductive cycle to capture potential effects on offspring.

New laboratory techniques can provide additional insights into pharamcokinetics and mechanisms. Use of xenografts of human tissue in rodents has allowed effects of drugs on specific tissues to be studied in a more physiological manner than in *in vitro* studies ^18^. Cell and tissue culture methods, placental perfusion methods ^19^ and recently reported organoids ^20^ have the potential to elucidate placental transfer, metabolism and endocrine function, and the effects of drugs on these. “Organ on a chip” models may be useful to further explore the maternal and fetal contributions, and computational models can be used for simulations and data integration ^16^.

Studies involving pregnant women are feasible and necessary to close the evidence gap on pharmacokinetics for medications in pregnancy. Indeed, there are compelling arguments that routine exclusion of pregnant women from drug research is unethical, and responsible inclusion should be mandatory ^21^. Draft guidance from the FDA suggests that pregnant women may be included in pharmacokinetic studies if (i) there are sufficient data from pre-clinical and clinical studies (including non-pregnant women) to assess the potential risk to pregnant women and fetuses and (ii) the potential risk is minimal and the purpose of the research is to gather important information that cannot be otherwise obtained ^22^. Opportunistic study designs involve women who are already on treatment, thus separating the decision to initiate treatment from the decision to participate in a study. This helps avoid concerns over the ethics of exposing mothers and babies to unknown risks for the purpose of research ^5^. In longitudinal designs, best suited for medications taken over a long period of time, women can serve as their own controls with samples taken at different gestational time points. It is also possible to involve pregnant women who will not benefit from the medicine under investigation in pharmacokinetic studies by restricting administration to a very brief period and using different groups of participants to determine drug dynamics in different trimesters and post-partum.

Potential for improving knowledge around pregnancy prescribing lies in the exploitation of health data collected for clinical or administrative purposes. Population-based birth registers, such as those of the Nordic countries, have been a rich source of information on drug safety in pregnancy ^23^. Recording of all live births and stillbirths within the region covered by the registry is mandatory, and registers contain basic information on the mother, father and neonate. Linkage through the unique personal identity number to other databases can provide data on prescribed medications as well as health, education and social conditions. A limitation, however, is that not all registers capture spontaneous pregnancy losses and induced terminations of pregnancy ^13^. Other sources of routinely collected data include medical record databases, such as the UK Clinical Practice Research Datalink ^24^ containing de-identified patient data from primary care linked to other health datasets, representative samples of the population, and administrative databases, such as those of health insurers ^25^.

Advances in the field of medical informatics mean that there has been expansion in the amount, depth and variety of health-related data collected, alongside increased computational capability to effectively perform analyses across large datasets ^26^. Research questions can now be successfully interrogated across multiple databases ^27^. Development of large networks of observational databases with billions of data points has the potential to overcome challenges of limited sample size and lack of power ^28^. Appropriate design, methodology and sophisticated analysis techniques are, however, essential to account for heterogeneity between data sources and for missing and incorrect data and to control for important confounders, particularly confounding by indication ^26, 29^. Further advances are conceivable through supplementation of coded data with information obtained from unstructured or ‘free text’ of medical records using automated data extraction techniques ^30^, integration of data from personal devices and wearable technologies ^29^, and ‘crowd sourced’ pharmacoepidemiology through analysis of data from social media platforms ^31^. Success depends on coherent approaches with considerable infrastructure and investment and on remaining cognisant of regulations and privacy concerns to ensure public confidence in data use and reuse.

Policy and centralised support for research into medication in pregnant women will be key to capitalising on opportunities to improve prescribing in pregnancy. In many countries, the average maternal age and rates of obesity have risen and both increases are associated with a growing burden of co-morbidities and pregnancy complications requiring medication. On a global scale, maternal conditions are the leading cause of mortality in children under five ^32^ and are a major determinant of women’s health and wellbeing. Therefore, investment to develop new treatments for pregnant women and to optimise existing medications is desperately needed. The dearth of medicinal product development for pregnancy conditions was demonstrated in a 2009 report which showed that only 17 drugs were under active development for maternal health indications ^33^. This represented less than 3% of the cardiovascular drug development pipeline and was less than for a single rare disease like amyotrophic lateral sclerosis ( 34 drugs in development). Regulatory frameworks governing medicines for children in Europe and the US have been successful in boosting paediatric drug development, paediatric clinical trials, and information on paediatric medications ^34, 35^. In the US, the Treating for Two initiative of the Centers for Disease Control and Prevention aims to improve both the evidence base and guidance for safer medication use in pregnancy to inform decision making ^36^ while the Obstetric-Fetal Pharmacology Research Centers Network supported by the National Institute of Child Health and Human Development aims to improve the understanding of obstetric pharmacokinetics and pharmacodynamics through non-clinical, clinical and pharmacokinetic and pharmacogenetic studies ^37^. However, clinical studies in pregnancy relating to drug development, dosing and effectiveness are still a rarity. A 2013 study found that only five of 558 USA industry-sponsored drug trials were specifically designed for pregnant women, and pregnant women were excluded from 95% of phase IV trials ^38^. A 2016 systematic review reported that only 0.32% of all active registered clinical trials of medicinal products were pregnancy drug trials, a tiny minority of those (6%) had a specific primary outcome relating to maternal or fetal health, and even fewer (4.4%) included pre-planned pharmacokinetics ^39^. Without legislative incentives or mandate, pharmaceutical companies are likely to remain unwilling to engage in research for pregnant women, and academic institutions and researchers may remain cautious of doing so.

## Conclusions

There is an acute need to provide women with appropriate information to judge the risks and benefits of treatment whether that is pre-pregnancy (in consideration of an unanticipated or planned pregnancy), in pregnancy or when breastfeeding. Medicine use in pregnancy is ubiquitous. Studies consistently show that the majority of pregnant women are prescribed one or more medications in pregnancy, rates are 50 to 80% depending on the setting, and when over-the-counter treatments are included rates approach 100% ^1, 40– 43^. Data from the US show that woman are prescribed an average of 2.6 medications during pregnancy ^41^, and a study from Italy estimates this figure to be 4.6 medications per pregnant woman ^44^. Both mothers and clinical care givers overestimate the teratogenic risks of medications and may err on the side of caution in the absence of available and clear safety data ^45^. This denies women appropriate therapy, and inadequate maternal treatment of disease can jeopardise both the mother’s and the baby’s wellbeing. On the other hand, the vast majority of medications currently in use have not been studied in a way that would reveal moderate teratogenic risks, let alone effects on miscarriage, or more subtle effects on long-term outcomes. A presumption of safety means that effects of even well-established therapies remain unmeasured. Every healthcare provider should discuss the implications to pregnancy from medications that they prescribe to women of (or soon to be of) reproductive age. Healthcare providers and women should also be cognisant of the uncertainties in the evidence. Adequate investment and policy are needed to improve knowledge about pregnancy prescribing, increase public confidence and promote industry engagement to develop much-needed new treatments for obstetric conditions. Lack of advancement in this area is unacceptably failing women and their families.

## Abbreviations

FDA, US Food and Drug Administration

## References

[1] Daw JR Hanley GE Greyson DL: Prescription drug use during pregnancy in developed countries: a systematic review. *Pharmacoepidemiol Drug Saf.* 2011;20(9):895–902. 10.1002/pds.2184 21774029 PMC3423446

[2] Glover DD Amonkar M Rybeck BF: Prescription, over-the-counter, and herbal medicine use in a rural, obstetric population. *Am J Obstet Gynecol.* 2003;188(4):1039–45. 10.1067/mob.2003.223 12712107

[3] McCormack SA Best BM: Obstetric Pharmacokinetic Dosing Studies are Urgently Needed. *Front Pediatr.* 2014;2:9. 10.3389/fped.2014.00009 24575394 PMC3920104

[4] Adam MP Polifka JE Friedman JM: Evolving knowledge of the teratogenicity of medications in human pregnancy. *Am J Med Genet C Semin Med Genet.* 2011;157C(3):175–82. 10.1002/ajmg.c.30313 21766440

[5] Sheffield JS Siegel D Mirochnick M: Designing drug trials: considerations for pregnant women. *Clin Infect Dis.* 2014;59 Suppl 7:S437–S444. 10.1093/cid/ciu709 25425722 PMC4303056

[6] Fisk NM Atun R: Systematic analysis of research underfunding in maternal and perinatal health. *BJOG.* 2009;116(3):347–56. 10.1111/j.1471-0528.2008.02027.x 19187366

[7] Kmietowicz Z: Women are unaware of pregnancy risks linked with sodium valproate. *BMJ.* 2016;355:i5829. 10.1136/bmj.i5829 27799146

[8] Sen A Nashef L: New regulations to cut valproate-exposed pregnancies. *Lancet.* 2018;392(10146):458–60. 10.1016/S0140-6736(18)31672-6 30129447

[9] Pariente G Leibson T Carls A: Pregnancy-Associated Changes in Pharmacokinetics: A Systematic Review. *PLoS Med.* 2016;13(11):e1002160. 10.1371/journal.pmed.1002160 27802281 PMC5089741

[10] Anderson GD: Pregnancy-induced changes in pharmacokinetics: a mechanistic-based approach. *Clin Pharmacokinet.* 2005;44(10):989–1008. 10.2165/00003088-200544100-00001 16176115

[11] Mitchell AA: Systematic identification of drugs that cause birth defects--a new opportunity. *N Engl J Med.* 2003;349(26):2556–9. 10.1056/NEJMsb031395 14695418

[12] Charlton RA Cunnington MC de Vries CS: Data resources for investigating drug exposure during pregnancy and associated outcomes: the General Practice Research Database (GPRD) as an alternative to pregnancy registries. *Drug Saf.* 2008;31(1):39–51. 10.2165/00002018-200831010-00004 18095745

[13] Charlton R de Vries, C: Systematic overview of data sources for drug safety in pregnancy research.2016 Reference Source

[14] Thorpe PG Gilboa SM Hernandez-Diaz S: Medications in the first trimester of pregnancy: most common exposures and critical gaps in understanding fetal risk. *Pharmacoepidemiol Drug Saf.* 2013;22(9):1013–8. 10.1002/pds.3495 23893932 PMC3996804

[15] Cragan JD: Medication use during pregnancy. *BMJ.* 2014;349:g5252. 10.1136/bmj.g5252 25150302

[16] Riley LE Cahill AG Beigi R: Improving Safe and Effective Use of Drugs in Pregnancy and Lactation: Workshop Summary. *Am J Perinatol.* 2017;34(8):826–32. 10.1055/s-0037-1598070 28142152 PMC6193221

[17] Al Jishi T Sergi C: Current perspective of diethylstilbestrol (DES) exposure in mothers and offspring. *Reprod Toxicol.* 2017;71:71–7. 10.1016/j.reprotox.2017.04.009 28461243

[18] van den Driesche S Macdonald J Anderson RA: Prolonged exposure to acetaminophen reduces testosterone production by the human fetal testis in a xenograft model. *Sci Transl Med.* 2015;7(288):288ra80. 10.1126/scitranslmed.aaa4097 25995226 PMC5044981

[19] Hutson JR Garcia-Bournissen F Davis A: The human placental perfusion model: A systematic review and development of a model to predict in vivo transfer of therapeutic drugs. *Clin Pharmacol Ther.* 2011;90(1):67–76. 10.1038/clpt.2011.66 21562489

[20] Turco MY Gardner L Kay RG: Trophoblast organoids as a model for maternal-fetal interactions during human placentation. *Nature.* 2018;564(7735):263–7. 10.1038/s41586-018-0753-3 30487605 PMC7220805

[21] White A: Accelerating the paradigm shift toward inclusion of pregnant women in drug research: Ethical and regulatory considerations. *Semin Perinatol.* 2015;39(7):537–40. 10.1053/j.semperi.2015.08.008 26385413

[22] (CDER) USDoHaHSFaDACfDEaR: Guidance for Industry Pharmacokinetics in Pregnancy — Study Design, Data Analysis, and Impact on Dosing and Labeling.2004 Reference Source

[23] Langhoff-Roos J Krebs L Klungsøyr K: The Nordic medical birth registers--a potential goldmine for clinical research. *Acta Obstet Gynecol Scand.* 2014;93(2):132–7. 10.1111/aogs.12302 24237585

[24] UK Clinical Practice Research Datalink 2018. Reference Source

[25] Hennessy S: Use of health care databases in pharmacoepidemiology. *Basic Clin Pharmacol Toxicol.* 2006;98(3):311–3. 10.1111/j.1742-7843.2006.pto_368.x 16611207

[26] Bate A Reynolds RF Caubel P: The hope, hype and reality of Big Data for pharmacovigilance. *Ther Adv Drug Saf.* 2018;9(1):5–11. 10.1177/2042098617736422 29318002 PMC5753994

[27] Bateman BT Heide-Jørgensen U Einarsdóttir K: β-Blocker Use in Pregnancy and the Risk for Congenital Malformations: An International Cohort Study. *Ann Intern Med.* 2018;169(10):665–73. 10.7326/M18-0338 30326014 PMC6854680

[28] Trifirò G Coloma PM Rijnbeek PR: Combining multiple healthcare databases for postmarketing drug and vaccine safety surveillance: why and how? *J Intern Med.* 2014;275(6):551–61. 10.1111/joim.12159 24635221

[29] Trifirò G Sultana J Bate A: From Big Data to Smart Data for Pharmacovigilance: The Role of Healthcare Databases and Other Emerging Sources. *Drug Saf.* 2018;41(2):143–9. 10.1007/s40264-017-0592-4 28840504

[30] Wu H Toti G Morley KI: SemEHR: A general-purpose semantic search system to surface semantic data from clinical notes for tailored care, trial recruitment, and clinical research. *J Am Med Inform Assoc.* 2018;25(5):530–7. 10.1093/jamia/ocx160 29361077 PMC6019046

[31] Golder S Chiuve S Weissenbacher D: Pharmacoepidemiologic Evaluation of Birth Defects from Health-Related Postings in Social Media During Pregnancy. *Drug Saf.* 2019;42(3):389–400. 10.1007/s40264-018-0731-6 30284214 PMC6426821

[32] WHO: Global Health Observatory (GHO) data: Causes of child mortality. New York;2017 Reference Source

[33] Fisk NM Atun R: Market failure and the poverty of new drugs in maternal health. *PLoS Med.* 2008;5(1):e22. 10.1371/journal.pmed.0050022 18215109 PMC2211556

[34] State of Paediatric Medicines in the EU 10 years of the EU Paediatric Regulation. European Commission;2017 Reference Source

[35] Ren Z Zajicek A: Review of the Best Pharmaceuticals for Children Act and the Pediatric Research Equity Act: What can the obstetric community learn from the pediatric experience? *Semin Perinatol.* 2015;39(7):530–1. 10.1053/j.semperi.2015.08.006 26455383 PMC4634862

[36] Treating for Two United States the Center for Disease Control and Prevention. Reference Source

[37] Obstetric-Fetal Pharmacology Research Centre Network National Institute of Child Health and Human Development. Reference Source

[38] Shields KE Lyerly AD: Exclusion of pregnant women from industry-sponsored clinical trials. *Obstet Gynecol.* 2013;122(5):1077–81. 10.1097/AOG.0b013e3182a9ca67 24104789

[39] Scaffidi J Mol BW Keelan JA: The pregnant women as a drug orphan: a global survey of registered clinical trials of pharmacological interventions in pregnancy. *BJOG.* 2017;124(1):132–40. 10.1111/1471-0528.14151 27297096

[40] Irvine L Flynn RW Libby G: Drugs dispensed in primary care during pregnancy: a record-linkage analysis in Tayside, Scotland. *Drug Saf.* 2010;33(7):593–604. 10.2165/11532330-000000000-00000 20553060

[41] Mitchell AA Gilboa SM Werler MM: Medication use during pregnancy, with particular focus on prescription drugs: 1976-2008. *Am J Obstet Gynecol.* 2011;205(1):51.e1–51.e8. 10.1016/j.ajog.2011.02.029 21514558 PMC3793635

[42] Ventura M Maraschini A D'Aloja P: Drug prescribing during pregnancy in a central region of Italy, 2008-2012. *BMC Public Health.* 2018;18(1):623. 10.1186/s12889-018-5545-z 29764430 PMC5952470

[43] Lupattelli A Spigset O Twigg MJ: Medication use in pregnancy: a cross-sectional, multinational web-based study. *BMJ Open.* 2014;4(2):e004365. 10.1136/bmjopen-2013-004365 24534260 PMC3927801

[44] Lupattelli A Spigset O Nordeng H: Adherence to medication for chronic disorders during pregnancy: results from a multinational study. *Int J Clin Pharm.* 2014;36(1):145–53. 10.1007/s11096-013-9864-y 24162929

[45] Widnes SF Schjøtt J: Risk perception regarding drug use in pregnancy. *Am J Obstet Gynecol.* 2017;216(4):375–8. 10.1016/j.ajog.2016.12.007 27988271

